# Low dose radiation prevents doxorubicin-induced cardiotoxicity

**DOI:** 10.18632/oncotarget.23013

**Published:** 2017-12-07

**Authors:** Xin Jiang, Yaqiong Hong, Di Zhao, Xinxin Meng, Lijing Zhao, Yanwei Du, Zan Wang, Yan Zheng, Lu Cai, Hongyu Jiang

**Affiliations:** ^1^ Department of Health Examination Center, The First Hospital of Jilin University, Changchun, Jilin 130021, China; ^2^ The School of Basic Medicine, Jilin University, Changchun, Jilin 130021, China; ^3^ Changchun University of Chinese Medicine, Changchun, Jilin 130021, China; ^4^ Department of Internal Neurology, The First Hospital of Jilin University, Changchun, Jilin 130021, China; ^5^ Department of Gerontology, The First Hospital of Jilin University, Changchun, Jilin 130021, China; ^6^ Pediatric Research Institute, The Departments of Pediatrics, Radiation Oncology, Pharmacology and Toxicology, The University of Louisville, Louisville, KY 40202, USA

**Keywords:** low dose radiation, hormesis, adaptive response, doxorubicin, oxidative stress

## Abstract

This study aimed to develop a novel and non-invasive approach, low-dose radiation (LDR, 75 mGy X-rays), to prevent doxorubicin (DOX)-induced cardiotoxicity. BALB/c mice were randomly divided into five groups, Control, LDR (a single exposure), Sham (treated same as LDR group except for irradiation), DOX (a single intraperitoneal injection of DOX at 7.5 mg/kg), and LDR/DOX (received LDR and 72 h later received DOX). Electrocardiogram analysis displayed several kinds of abnormal ECG profiles in DOX-treated mice, but less in LDR/DOX group. Cardiotoxicity indices included histopathological changes, oxidative stress markers, and measurements of mitochondrial membrane permeability. Pretreatment of DOX group with LDR reduced oxidative damages (reactive oxygen species formation, protein nitration, and lipid peroxidation) and increased the activities of antioxidants (superoxide dismutase and glutathione peroxidase) in the heart of LDR/DOX mice compared to DOX mice. Pretreatment of DOX-treated mice with LDR also decreased DOX-induced cardiac cell apoptosis (TUNEL staining and cleaved caspase-3) and mitochondrial apoptotic pathway (increased p53, Bax, and caspase-9 expression and decreased Bcl2 expression and ΔΨm dissipation). These results suggest that LDR could induce adaptation of the heart to DOX-induced toxicity. Cardiac protection by LDR may attribute to attenuate DOX-induced cell death via suppressing mitochondrial-dependent oxidative stress and apoptosis signaling.

## INTRODUCTION

Doxorubicin (DOX) is an effective drug commonly used to treat both solid tumors (breast cancer, carcinomas) and hematologic malignancies (leukemia, lymphomas) [1]. The broad usage of DOX for over 50 years has dramatically improved cancer survival statistics. However, the dose-dependent cardiotoxicity compromises its clinical usage [2–4]. With the increasing population of cancer survivors, there is a growing need to elucidate the mechanisms for DOX-induced cardiomyopathy and the potential ways to prevent its development and progression. The appreciated mechanisms for DOX-induced cardiomyopathy include oxidative stress, inflammation, dysregulation of calcium handling and cellular contractility, mitochondrial degeneration, and apoptotic cardiomyocyte death [2–5]. However, oxidative stress is the major contributor in triggering and progressing DOX-induced myocardial biochemical and pathological changes, leading to the final structural remodeling and dysfunction [2, 6].

Based on the concept that free radicals involve in DOX cardiotoxicity, a number of antioxidant compounds were tested in animal models and cancer patients [7–9]. Studies with animal models of cardiac overexpression of certain antioxidants such as catalase and metallothionein have shown the well protection from DOX-induced cardiotoxicity [7, 8]. To date, despite Dexrazoxane, an intracellular iron chelator to reduce the potential generation of iron-derived free radicals, has been approved as a prophylactic medication used in clinical practice for the cancer patients who received DOX treatment [10], the potentially hematological toxicities of Dexrazoxane promotes remains to further search for a better approach that can ameliorate DOX cardiotoxicity without compromising its antineoplastic activity [10]. Current two major concerns include: (a) Exogenous administration of antioxidants could not maintain consistent levels between two dosing [10–13]; therefore, it may be a better way to induce endogenous cardiac antioxidants; (b) Systemic administration of any drug may not only protect against DOX cardiac toxicity, but also reduce the efficacy of DOX cancer therapy [10–13]; therefore, any non-invasive approach that can specifically stimulate cardiac antioxidant capacity may be a better strategy to prevent DOX cardiac toxicity without impact on its cancer therapeutic effects.

We proposed the application of low-dose radiation (LDR) to protect the heart from DOX since LDR could prevent damage to normal tissue induced by radiation and anticancer drugs [14–16]. LDR, which is ubiquitous in our environment, is defined as a radiation dose of 100 mSv or less (≤100 mGy). Low-dose-rate radiation is defined as the rate of radiation exposure at 6 mSv or less per hour (<6 mSv/h). For low linear energy transfer (LET) radiation, 1 Gy (exposure dose) is equal 1 Sv (absorbed dose) (see references in review [17]. Distinct from high-dose radiation that causes cytotoxic effects *in vitro* and *in vivo*, LDR induces an adaptive or hormetic response in cells and tissues, showing a tolerance to subsequently high dose of radiation- or chemical-induced damage *in vitro* and *in vivo* [14–16]. Our previous study suggested that LDR stimulated growth of normal cells but not leukemia or solid tumor cells *in vitro* and LDR also did not stimulate growth of solid tumor cells *in vivo* [16]. In addition, LDR decreases cancer risks via stimulating anticancer immunity [18–20]. The protective effects of LDR were attributed to up-regulated antioxidants [21] and DNA repair enzymes [22, 23]. We already demonstrated the protective effect of LDR on various diabetic complications, such as the testicular, renal, and cardiac damages in diabetic rats and mice [24–26]. Therefore, the distinct features of LDR, compared to other compounds discussed above [9–13], include: (a) A non-invasive approach; (b) Applicable to heart area without affecting other organs; (c) Ability to stimulate multiple defense functions; (d) No detectable and obvious side-effect.

In fact, pre-chemotherapeutic LDR was able to alleviate anticancer drug, cyclophosphamide-induced damaging effects on the liver [14]. However, there was no study that has explored whether LDR could provide protective effect against DOX cardiotoxicity *in vivo*. Therefore, the present study was aimed to develop a novel and non-invasive approach, LDR (75 mGy X-rays), to prevent DOX cardiotoxicity in mice. Considering the fact that LDR is able to attenuate oxidative stress, mitochondrial dysfunction and apoptosis in a rat model of Parkinson’s diseases [27, 28], we also examined the cardiac mitochondria and associated oxidative stress and cell death in order to elucidate the underlying mechanisms by which LDR protects the heart from DOX.

## RESULTS

### Effect of LDR on DOX-induced cardiotoxicity

**Figure 1:**
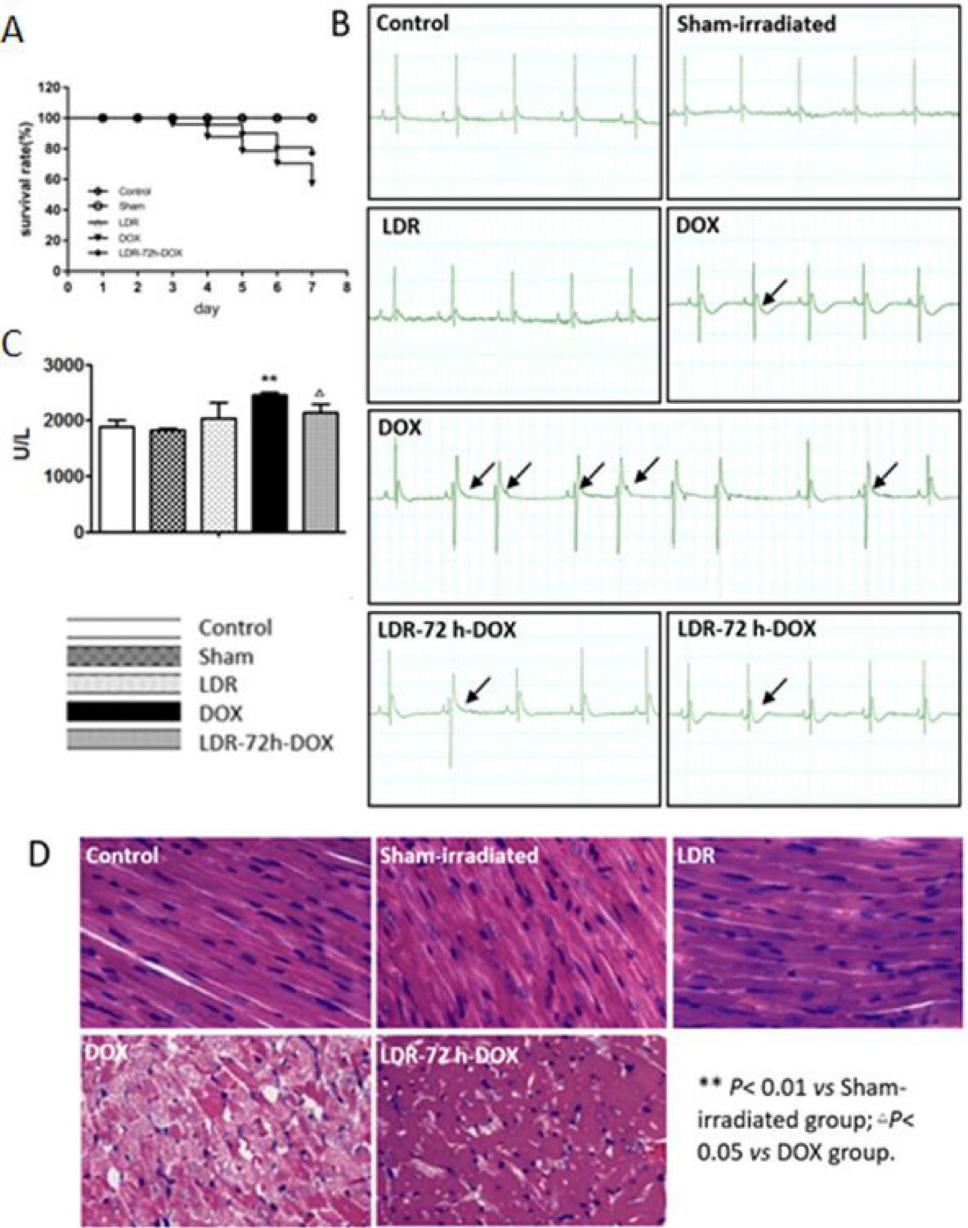
Effect of LDR on DOX-induced animal mortality and cardiotoxicity (**A**) Survival rate of the mice in each group at the 5th day after the last DOX injection (*n =* 10–20). (**B**) Representative ECG results that show several kinds of ECG changes including arrhythmia, T wave flat, and ST-segment depression in DOX-treated mice. (**C**) LDH activities among different groups of mice. (**D**) Histopathological changes (×100), showing myofibrillar disarrangement and vacuolization of the cytoplasm in the heart of DOX-treated mice.

To test whether LDR can attenuate DOX-induced cardiac toxicity we observed the general appearance of the mice, animal survival rate, and changes of ECG. DOX-treated mice were sick and weak, and their fur became scruffy. These changes were less pronounced in LDR/DOX mice. There was no difference for their appearance among control, sham and LDR mice. The mice in control, sham-irradiated, and LDR groups were 100% survived, whereas mice in the DOX group showed 80% and 60% survival at the 6th and 7th day after treatment. Pre-exposure of DOX-treated mice to LDR increased animal survival rate to 90% and 80% survival rate at the 6th and 7th days after DOX treatment, respectively (Figure 1A).

Electrocardiogram (ECG) analysis showed normal profiles in the control and LDR groups. However, there are several kinds of abnormal ECG profiles in DOX-treated mice, which were frequently seen in DOX alone group with several kinds of abnormalities, compared to LDR/DOX group. The abnormal ECG changes include arrhythmia, T wave flat, and ST-segment depression. The typical changes of ECG of animals were shown in Figure 1B.

Since increased serum lactate dehydrogenase (LDH) has been often used as the main serum biochemical marker of myocardial damage [29], we further examined the protective effect of LDR pretreatment on DOX-induced cardiotoxicity by detecting serum levels of LDH. As shown in Figure 1C, there was no significant difference of the enzyme levels among the control, the sham-irradiated, and the LDR groups. However, the level of serum LDH was significantly higher in DOX group as compared with that in Sham-irradiated group while pretreatment with LDR attenuated DOX-induced elevation of LDH serum level.

Morphological changes in the heart were also examined by H&E staining (Figure 1D), which showed that the histology of the heart tissue from control and sham-irradiated mice showed normal morphological appearances, whereas in DOX group, myofibrillar disorder and vacuolization of the cytoplasm were observed. The histology of heart tissues from LDR+DOX group showed less myofibrillar disorder and vacuolization of the cytoplasm. Compared with the mice in control group, there was no significant change of myocardial histopathology of mice in LDR group (Figure 1D). These results indicated that DOX can cause significant cardiac toxicity, and LDR effectively resisted DOX-induced cardiotoxicity.

### Effect of LDR on DOX-induced myocardial cell death

**Figure 2:**
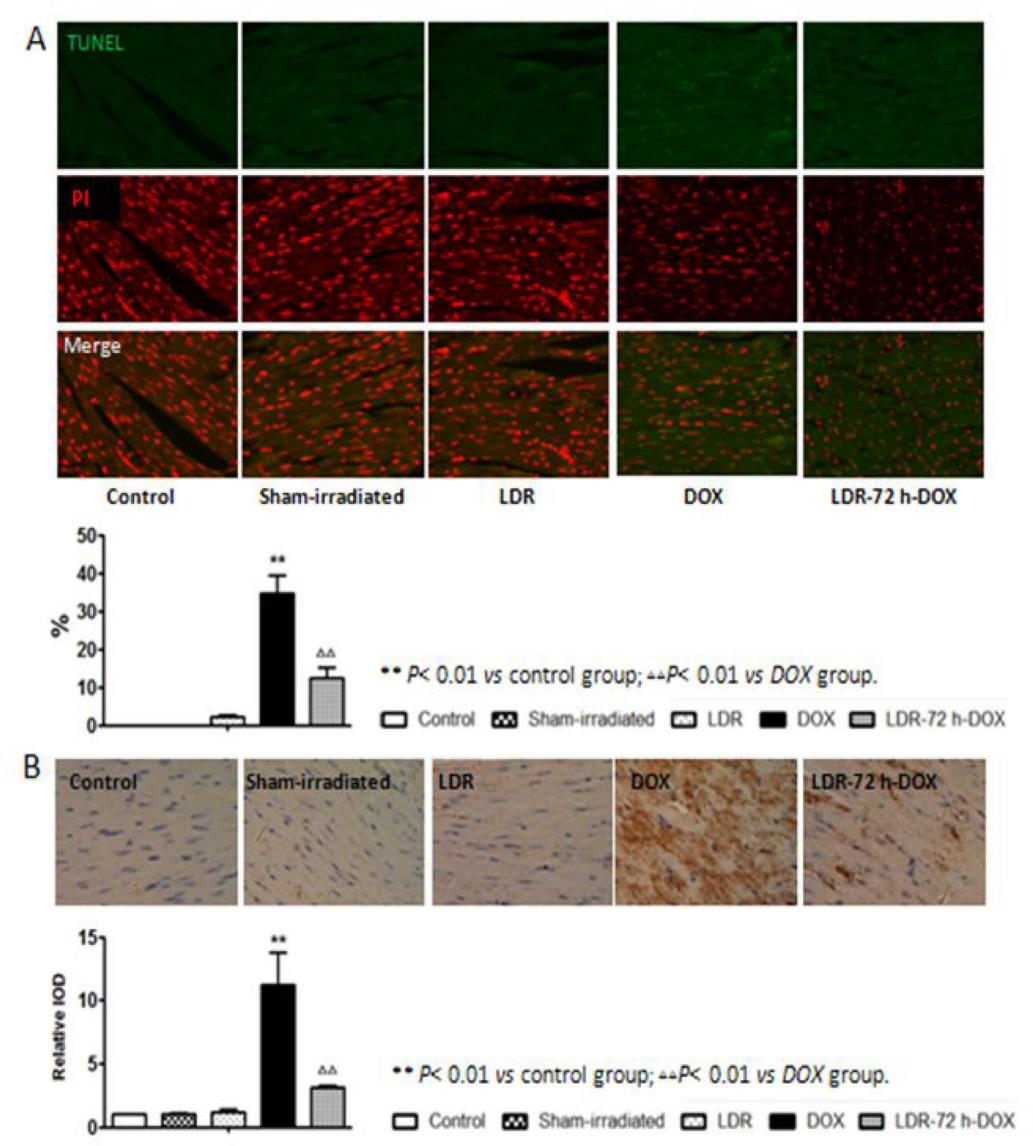
Effect of LDR on DOX-induced myocardial apoptosis **(A**) Apoptotic cells were measured by TUNEL assay in which green fluorescence indicates apoptosis, and quantitatively presented as apoptotic index (AI), e.g.: the percentage of TUNEL positive cells. (**B**) Expression of the cleaved caspase-3 was determined by immunohistochemical staining (200×), where brown color (positive) and light blue color (negative) denotes the cell expressing with or without cleaved caspase-3, respectively. Expression of cleaved caspase-3 was quantitatively analyzed as integrated optical densities (IOD) and presented as relative IOD in experimental group to that of control. Values are expressed as means ± SE (*n* = 6).

Accumulated evidence showed that DOX can cause apoptotic cell death in the animal heart [5, 11, 12]. To test whether LDR-alleviated DOX cardiotoxicity was associated with the reduction of apoptosis we performed TUNEL staining in which green fluorescence indicates apoptosis as shown in Figure 2A. Quantitative analysis for the apoptotic index (AI) shows no or few cells with green fluorescence in the control, sham-irradiated, and LDR groups. However, intense green fluorescence was observed in DOX group whereas less green fluorescence was seen in LDR/DOX group (*P <* 0.01).

**Figure 3:**
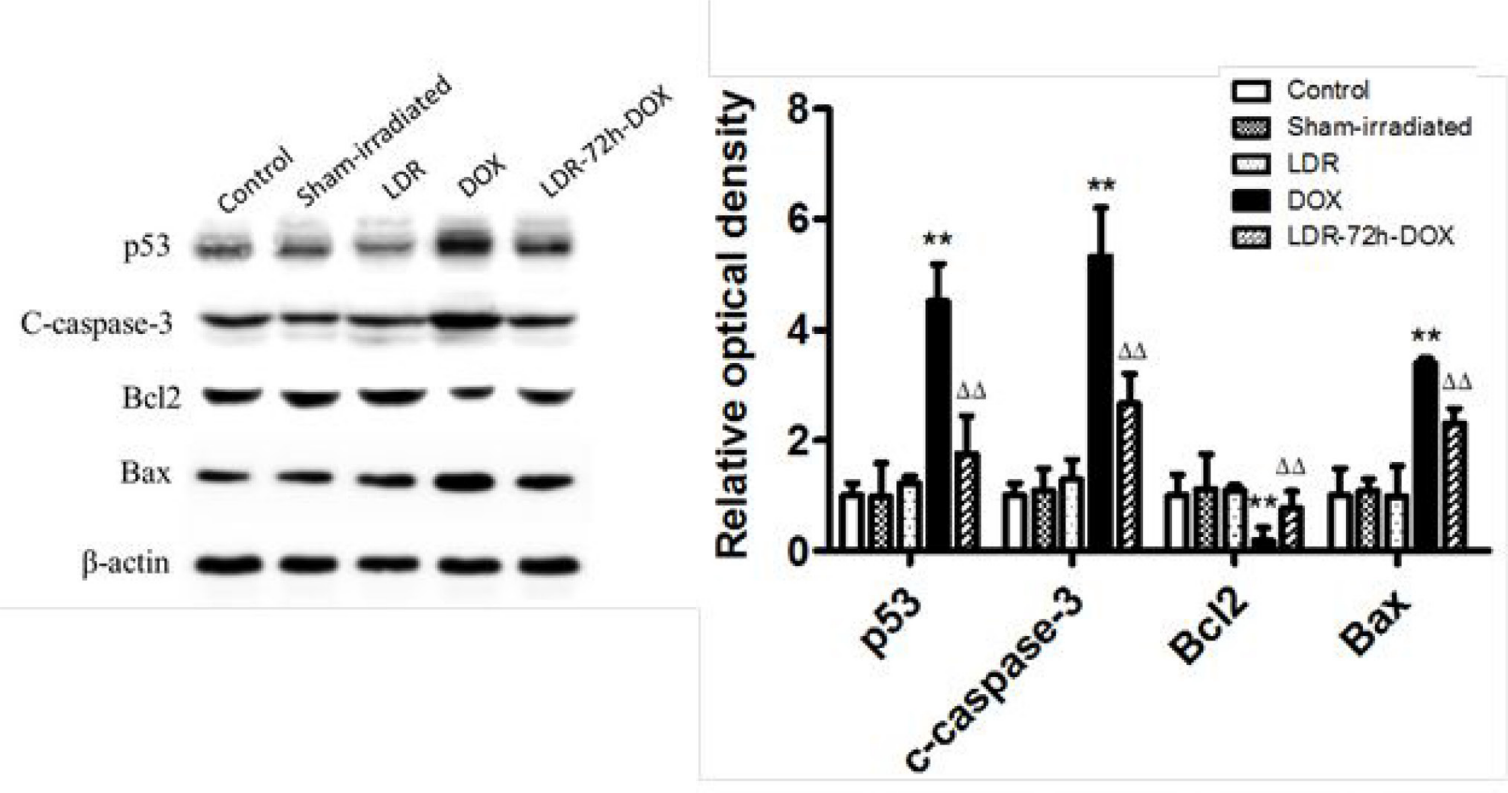
Western blots for the effect of LDR on DOX-induced p53 and mitochondrial apoptotic signaling Expression of p53, cleaved caspase-3 (C-capase-3), Bcl2, and Bax protein was examined with Western blots, followed by quantitatively densitometric analysis of the target protein bands. The relative optical density (target protein band density to β-actin value) was presented as means ± SE (*n* = 6). **p < 0.01 vs Sham-irradiated group, ^∆∆^*p <* 0.01 vs DOX group.

Next the expressing level of cleaved caspase-3, a key mediator of apoptosis, was examined by immunohistochemical staining as shown in Figure 2B, followed by semi-quantitative analysis, show very low level of cleaved caspase-3 among the control, sham-irradiated, and LDR groups, but DOX induces a significant increase in cleaved caspase-3 level, which was remarkable prevented by pre-LDR treatment (Figure 2B). The TUNEL positive cells and cleaved caspase-3 analyses clearly suggested the induction of apoptotic cell death by DOX and DOX-induced apoptotic cell death is significantly prevented by pre-exposure to LDR. The immunohistochemical staining results of cleaved caspase was further confirmed by Western blot analysis (Figure 3).

### Effect of LDR on DOX-induced mitochondria-dependent apoptotic pathway

It was reported that apoptotic markers, such as caspase activation, were detected after 24 h of DOX treatment and Bax and p53 translocation to mitochondria as well as the formation of Bax clusters in the cytosol [30]. Therefore, we tried to ensure whether LDR-mediated protection from DOX-induced apoptosis is attributed to its prevention of mitochondria-dependent apoptotic pathway. First we examined and confirmed the increased expression of p53 in DOX-treated mice in parallel with the increase in cleaved capaspase-3 (Figure 3), both which were prevented by pre-exposure of DOX-treated cells to LDR.

**Figure 4:**
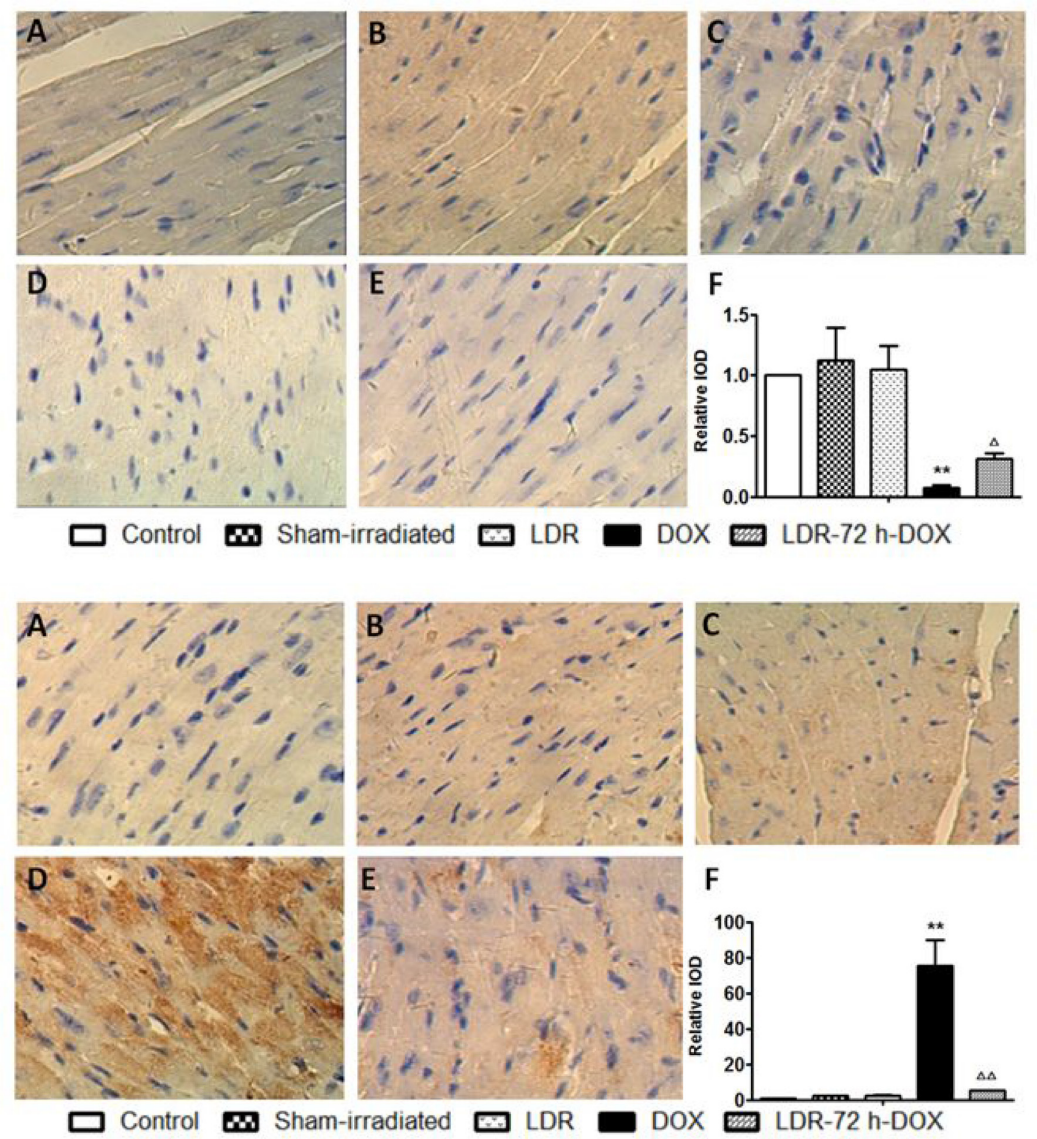
Effect of LDR on DOX-induced mitochondria-dependent apoptotic pathway Expression of Bcl-2 (Top panel) and Bax (Bottom panel) by immunohistochemical staining (200×) was quantitatively analyzed for IOD. Values are expressed as means ± SE (*n* = 6). The IOD was normalized to the Control.

Second we examine mitochondrial Bcl2 family anti- and pro-apoptotic members: Bcl2 and Bax protein expression with Western blotting assay, which showed no much change for the expression of either Bcl-2 or Bax protein among groups of control, sham-irradiated and LDR mice (Figure 3). However, significantly decreased expression of Bcl2 and increased expression of Bax were seen in DOX group, but not in LDR/DOX group. The Western blotting results were supported by immunohistochemical staining for both Bcl-2 and Bax expressions, followed by semi-quantitative analysis (Figure 4).

**Figure 5:**
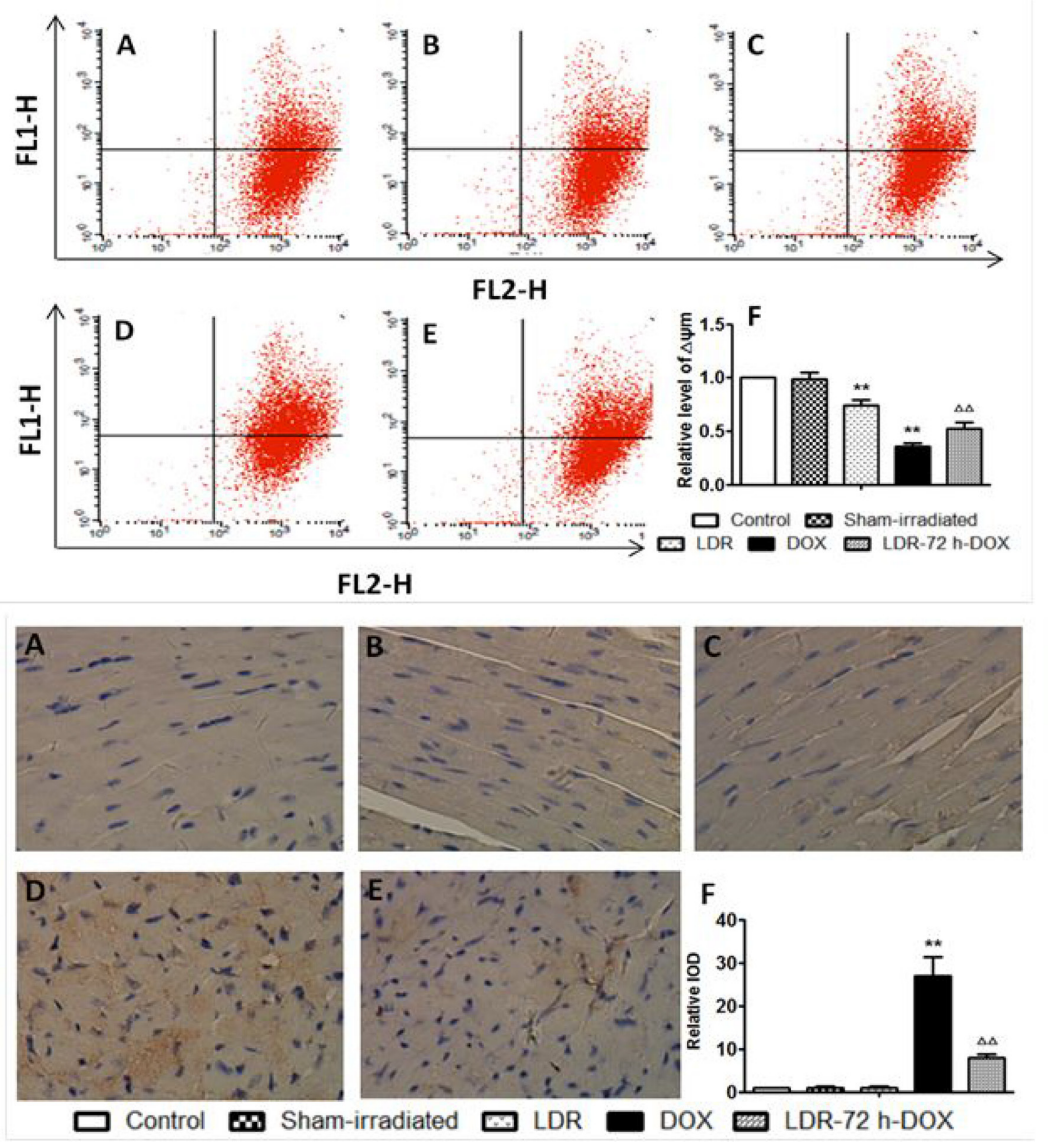
Effect of LDR on DOX-induced mitochondria-dependent apoptotic pathway (**A**) Representative profile of flow-cytometry and quantitative analysis of the fluorescence intensity of cardiac myocytes after staining with JC-1, measured with flow cytometry. (**B**) Expression of cleaved caspase-9 by immunohistochemical staining (200×). Quantitative image analysis for immunohistochemical staining expressed as IOD. Values are expressed as means ± SE (*n* = 6). The IOD was normalized to the Control.

Above assays indicated the involvement of mitochondrial apoptosis pathway in the induction of cardiac cell death by DOX, which promoted us to further detect mitochondrial membrane potential (ΔΨm) with flow cytometry (Figure 5 Top panel). No difference was found for ΔΨm of the hearts between control and sham groups, but ΔΨm in the DOX group was significantly lower than that of the sham group. DOX-decreased ΔΨm level was significantly improved by pre-exposure to LDR in LDR/DOX group (Figure 5 Top panel).

Caspase-9 as a key mediator for mitochondrial apoptosis pathway was also examined with immuno-histochemistry, followed by semi-quantitative analysis (Figure 5 bottom panel). There was no much positive staining among groups of the control, sham and LDR mice, but there was an increase in caspase-9 expression in DOX group but not in LDR/DOX group.

### Effect of LDR on DOX-induced oxidative stress

**Figure 6:**
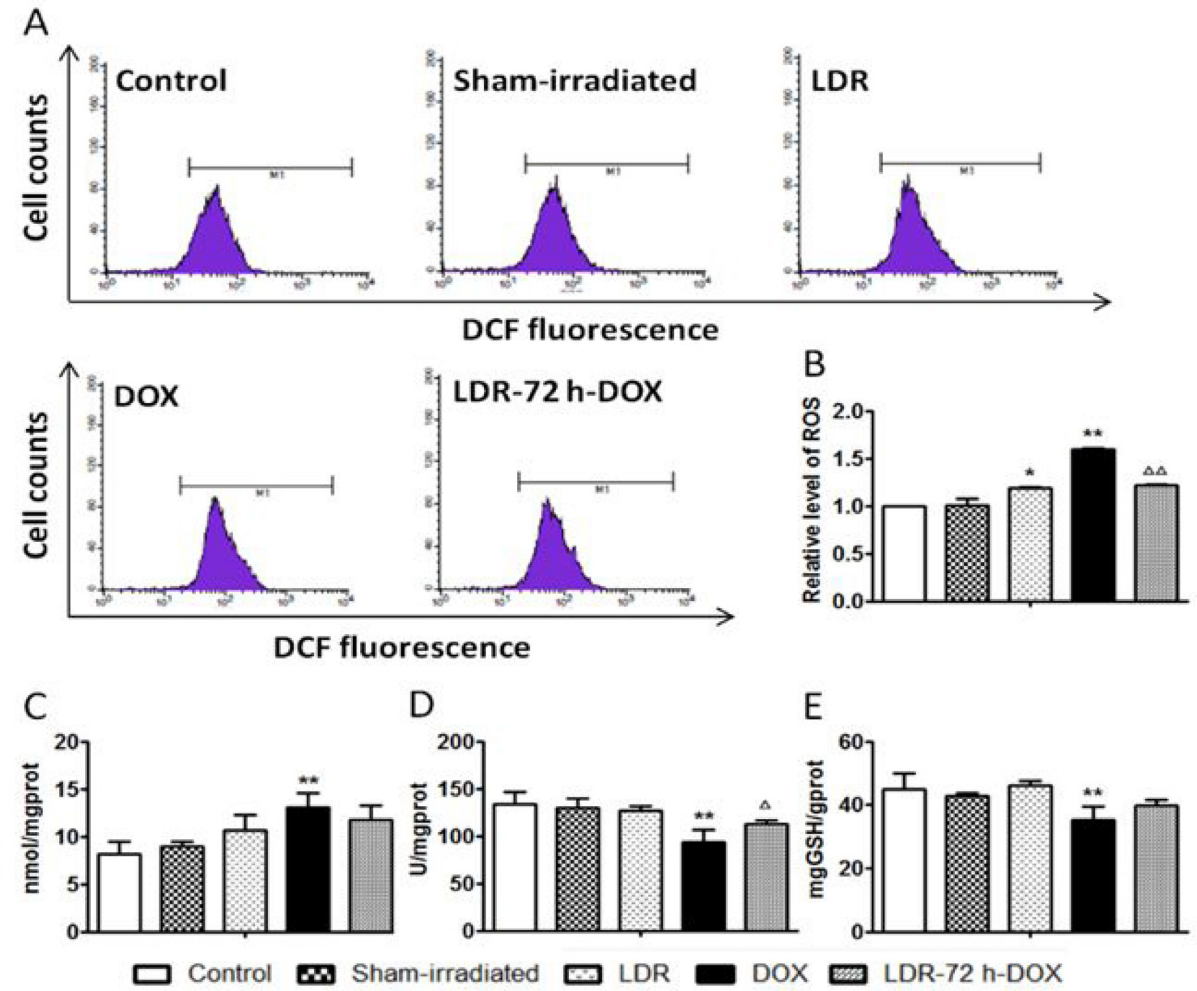
Effect of LDR on DOX-induced oxidative stress The concentration of intracellular H_2_O_2_ and other ROSs was measured by highly fluorescent DCFH-DA with flow cytometry, and expressed as flow cytometric profiles (**A**) and graphical columns (**B**). The level of MDA as lipid peroxidation was measured (**C**). The activities of both SOD (**D**) and GSH-PX (**E**) were also measured with corresponding assay kits. Values are expressed as mean ± SE (*n =* 6), *P < 0.05, ** *P <* 0.01 vs Sham group; ^∆^*P <* 0.05, ^∆∆^*P <* 0.01 vs DOX group.

Reportedly DOX-triggered the mitochondria-dependent apoptotic pathway in cardiomyocytes is predominantly attributed to DOX-induced oxidative stress [6–9, 31]; accordingly, we evaluated the level of ROS by flow cytometry with the probe of cell permeant DCFH-DA to measure primarily H_2_O_2_ and other ROS. As shown in Figure 6A, 6B, the level of ROS increased significantly in the DOX group compared with the Sham group (*P <* 0.01), whereas the increase by DOX was attenuated by LDR pretreatment. Compared with the Sham group, the level of ROS in LDR group was slightly increased (*P <* 0.05).

As one of oxidative damage index, lipid peroxidation level, shown by malondialdehyde (MDA), was examined (Figure 6C). The level of MDA was not significantly altered in LDR group but increased in DOX group compared with Sham group (*P <* 0.01). Pretreatment of DOX-treated mice with LDR slightly reduced DOX-increased MDA level.

We also measured the activities of SOD and GSH-PX (Figure 6D, 6E), which were both decreased in the DOX group compared to the control (*P <* 0.01), whereas DOX-decreased activities of SOD and GSH-PX were reversed by LDR pretreatment. These results suggested that LDR inhibition of DOX-induced oxidative stress and damage might be related to the up-regulated activity of antioxidant enzymes and reduced production of ROS.

**Figure 7:**
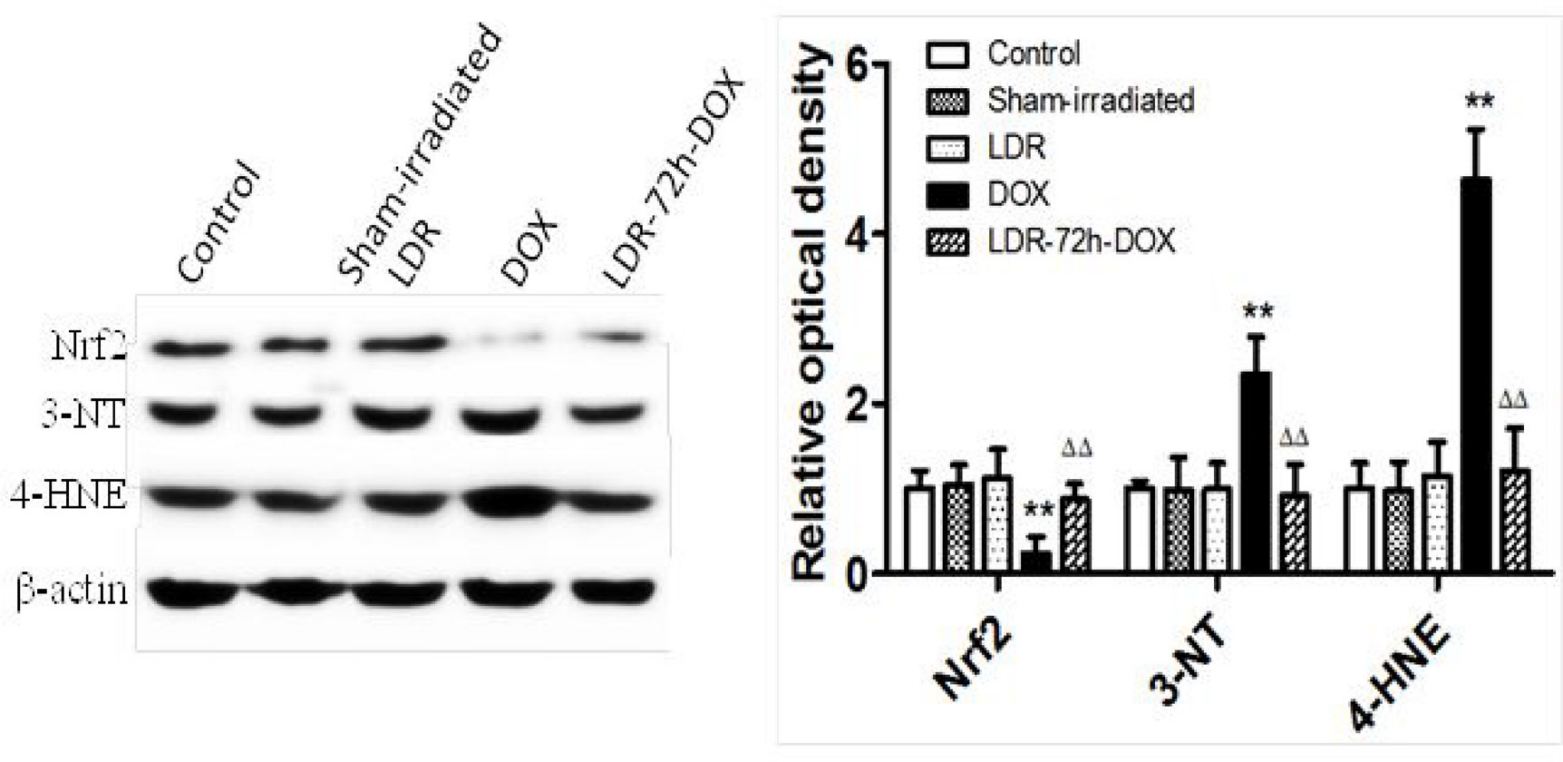
Effect of LDR on DOX-induced oxidative stress associated proteins Expression of Nrf2, 3-NT, and 4-HNE was analyzed with Western blots, followed by densitometric analysis of the target protein bands. The relative optical density (target protein band density to β-actin values) was presented as means ± SE (*n* = 6). **p < 0.01 vs Sham group, ^∆∆^*p <* 0.01 vs DOX group.

Using Western blotting assay, the expression of nuclear factor erythroid 2-related factor 2 (Nrf2) as transcription factor involved in cellular redox homeostasis was further examined along with other oxidative and nitrative variables, including 4-hydroxynonenal (4-HNE) and 3-nitrotyrosine formation (Figure 7). The heart of mice with LDR and sham-irradiation did not exhibit any change of Nrf2, 3-NT and 4-HNE levels; however, heart of mice treated by DOX alone exhibited a decrease in Nrf2 protein level along with increases in 3-NT and 4-HNE accumulation, which were almost completely attenuated by pre-exposure to LDR in LDR/DOX group.

## DISCUSSION

**Figure 8:**
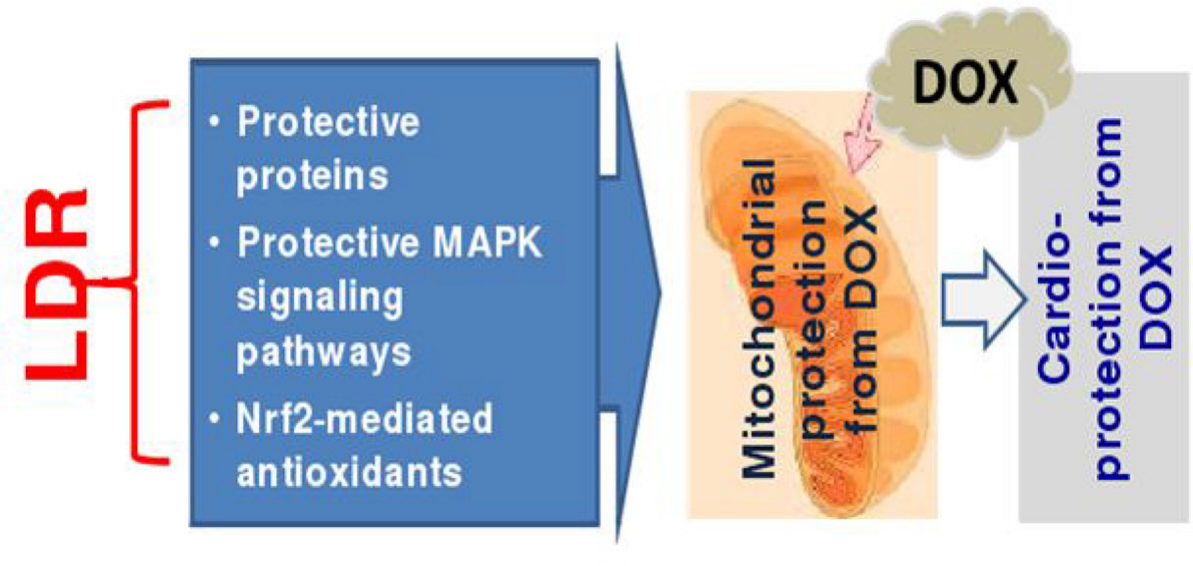
Hypothesized mechanisms by which LDR protects the heart from DOX Based on previous published work from the authors own and others, we assume that LDR may generate small amount of ROS (a) that stimulates cardiac cells to express protective proteins [21–23]; (b) oxidizes Keap1 to disassociate Nrf2 that translocates to nucleus where Nrf2 transcriptionally up-regulates its down-stream antioxidant genes [21, 40–42]; (c) stimulates ERK1/2 or ATM/Akt-mediated protective signaling pathways [21, 43]; All these possible mechanisms protect mitochondrial damage from DOX, resulting in the protection from DOX-induced cardiac cell death and cardiac toxicity.

DOX is a powerful antibiotic used to treat a multitude of human neoplasms; however, cardiac toxicity compromises its clinical applications [2–4]. Therefore, the search for a safe and effective approach to prevent or reverse DOX-induced cardiotoxicity remains a critical issue in both cardiology and oncology. Adaptive response or hormesis induced by LDR shows a protective effect on subsequent challenges-induced damage *in vitro* and *in vivo*. The present study confirms protective effects of LDR on DOX-induced cardiotoxicity, which may be associated with LDR-stimulated cardiac antioxidant capacity in reducing oxidative stress derived from mitochondria-dependent ROS/RNS generation, as outline in Figure 8.

In this study, we first evaluated the ameliorating effect of LDR on DOX-induced cardiotoxicity in mice. Previous studies have shown DOX-induced myocardial injuries in animal models and human biopsies, manifested by the significant elevation in activity of serum LDH [29] and damages such as cytoplasmic vacuolization and myofibrillar disorder [32, 33]. Consistent with these previous studies, here we also found DOX-induced cardiotoxicity, reflected by abnormal ECG profiles, increased serum LDH activity, and alterations of histopathology of the heart. However, we demonstrated the prevention of DOX-induced changes, including cardiac function, serum cardiac enzyme and structure by pre-exposure to LDR at 75 mGy.

Oxidative stress has been considered a major contributor in DOX-induced myocardial dysfunction [6–9, 31, 45–48]. To support this notion, mice with cardiac overexpression of catalase and metallothionein are resistant to DOX-induced cardiotoxicity [7, 8]. Induction of endogenous antioxidant with procyanidins, extracted from grape seeds also protected from DOX-induced cardiotoxicity [34]. Here we thus explored whether LDR protection against DOX-induced cardiotoxicity is associated with its prevention of oxidative stress and damage. We demonstrated that DOX-increased oxidative stress and damage, shown by increased ROS and oxidative damage are attenuated by pre-exposure to LDR in DOX-treated mice.

Reportedly DOX-induced apoptosis of cardiomyocytes as the most direct cause of DOX cardiotoxicity may be mitochondria-dependent [30, 31, 35]. Studies have shown that mitochondria-dependent apoptotic pathway in cardiomyocytes is mediated by DOX-induced oxidative stress [6–9, 31]. Oxidative damage to mitochondrial membranes, enzymes, and electron transport chain, which facilitates mitochondrial permeability transition pore opening, changes ^∆^Ψm, and releases cytochrome c to cytosol, resulting in the activation of caspase-9, to activate its effector caspases, such as caspase-3, to induce cell death [36, 37]. Mitochondria-mediated apoptosis is also modulated by the Bcl-2 family of proteins, including pro-apoptotic (e.g. Bax and Bid) and anti-apoptotic (e.g. Bcl-2 and Bcl-xL) members [30, 38]. Increasing the expression of Bcl-xL or Bcl-2 can protect against DOX-induced cardiotoxicity [30, 38]. Our study shows that DOX has profound effects on the Bcl-2 family proteins: down-regulating Bcl-2 and up-regulating Bax expression, which is in agreement with previous studies.

In addition, we here show the preventive effect of pre-exposure to LDR on DOX-induced decrease in mitochondrial permeability and increase in caspase-9 cleavage. Pre-exposure of DOX-treated mice with LDR attenuated DOX-increased ratio of Bax to Bcl-2 expression. A recent study reported that combined pre-LDR at 0.5 Gy γ-rays with sea cucumber or valsartan could alleviate DOX-induced cardiotoxicity via inhibiting oxidative stress and apoptosis [39]. However, the dose of 0.5 Gy is not typical LDR (∼100 mGy, see references in review [17]). In addition, there was also no comparison between DOX and LDR/DOX groups, since they gave rats DOX (2.5 mg/kg, ip) in six equal injections over a period of 2 weeks with and without either sea cucumber (14.4 mg/kg, p.o) or valsartan [30 mg/kg, p.o) for 8 successive weeks. LDR was only given once prior to the first dose of DOX. Therefore, the present study is the first one to show that DOX-induced cardiotoxicity is preventable by pre-exposure to LDT at 75 mGy low LET radiation such as X-rays.

Regarding the mechanisms by which LDR preserves mitochondrial integrity, we do not have direct evidence based on the present study; however we assumed the protective effects of LDR, as illustrated in Figure 8, most likely due to its following reasons: (1) LDR stimulates Akt and Nrf2 functions via generating small amount of ROS [21, 40] to up-regulate multiple antioxidants or free radical scavenging capacities [21, 24, 40]; (2) LDR-increased expression of mitochondrial superoxide dismutase [41, 42] which may be mediated by LDR-up-regulated Nrf2 transcription function, contributing to the resistance of cardiac cells to DOX; (3) LDR-stimulated mitogen activated protein kinase that turn on cell survival signaling in normal tissues, but not in tumor tissue [40, 43].

In the cells and tissues, small amount of ROS is necessary for cell mitogenic and proliferative functions, while intermediate levels of ROS cause either temporary or permanent cell growth arrest, such as replicative senescence, and severe oxidative stress due to excess ROS ultimately causes cell death [44, 45]. In the present study we found the slight increase in ROS amount in LDR group compared to control, but the remarkable increase in ROS amount in DOX group (Figure 6B). It is known that Keap 1, which is enriched in cysteine, promotes Nrf2 ubiquitin-mediated degeneration. Several compounds, such as sulforaphane (SFN), are able to generate small amount of ROS to oxidize Keap1 to release Nrf2, thereby stabilizing Nrf2 function [46, 47]. Therefore, we assume that LDR-generated small amounts of ROS may oxidize Keap1, thereby releasing and stabilizing Nrf2 that results in up-regulation of multiple antioxidants [21, 40]. However, although both SFN and LDR can generate small amount of ROS to stimulate Nrf2 function, exposure to LDR is non-invasive while SFN must be administrated systemically so that LDR remains the best approach. All these possibilities are summarized in Figure 8.

In summary, to use LDR for preventing cardiotoxicity caused by DOX cancer therapy has the following advantages or novelties: (a) It is a non-invasive approach to stimulate multiple endogenous protective mechanisms; (b) It can be given only cardiac region to stimulate cardiac adaptive or hormetic response without systemic stimulating effect, including cancer tissues. (c) Due to the different growth and metabolic features of tumor cells and normal tissue cells, we can also design LDR doses and interval times between LDR and DOX cancer therapy that all favorite to normal tissue to develop the resistance to DOX. For instance, we have demonstrated that LDR induced adaptive response in normal cells but not in tumor cells under certain conditions [16, 40]; (d) LDR is able to stimulates anti-tumor immunity in animal model [15, 18–20].

It should be noticed that a recent study reported that LDR at 300 mGy X-rays could increase ischemic limb perfusion recovery and capillary and collateral densities via inducing the expression of proangiogenic genes on mice [48]; since this study used a dose of 300 mGy, it is urgent to determine whether radiation at doses of less 200 mGy or even at the dose used in the present study (75 mGy X-rays) will have similar effects remains unclear, but really worthy to be explored. In addition, recently there was a case report that exposure to LDR via computed tomography (CT) scans at a range of 39–47 mGy X-rays was Alzheimer disease [49, 50]; In detail, a 81-year-old patient with Alzheimer disease was admitted to hospice with a life expectancy of less than 6 months, to be treated with CT scans once a while with the request by patient’s husband, and then the patient recovered sufficiently to be discharged from hospice on about four months later [49, 50]; however, more than 2 years after the initial treatment, the patient is in slow decline as described in a late response letter [51]. Although this is the first case that shows the beneficial effect on patient from CT scans, more clinical studies need to be explored for this great potential. Therefore, LDR may be a novel approach to prevent DOX-induced cardiotoxicity and enhance the effectiveness of cancer therapeutics via stimulating multiple functions in the heart without impact on tumors [15, 19, 21, 27, 51].

## MATERIALS AND METHODS

### Animals

BALB/c mice (female, weighting 18 ± 2g, aged 4–6 weeks) were provided by Animal Experiment Centre of Basic Medical Sciences, Jilin University The experimental protocol was approved by the Animal Ethics Review Committee of Basic Medical Sciences, Jilin University, in accordance with the regulations of the Institutional Committee for the Care and Use of Laboratory Animals. We selected female mice since DOX is mainly used in breast cancer chemotherapy [1]. The mice were maintained and housed under a 12/12 h light/dark cycle in air-conditioned rooms at 25 ± 2°C, with free access to food and water. Animals were accommodated for one week before experimentation.

### Drugs and chemicals

DOX was purchased from Shenzhen Main Luck Pharmaceuticals Inc. (Shenzhen, China). Caspase-9, Caspase-3, Bcl-2, Bax antibodies and secondary antibodies directed against rabbit or goat (HRP-conjugated anti-rabbit or anti-mouse antibody) were purchased from Proteintech Group (Chicago, IL, USA). ROS detection kit for flow cytometry was purchased from Enzo R Life Sciences (Farmingdale, NY, USA). Fluorometric TUNEL system was purchased from Promega (Madison, WI, USA). All chemicals and solvents were of analytical grade.

### Radiation conditions

A Philips deep X-ray machine was used with 200 kVp, 10 m A and filters of 0.5 mm copper plus 1.0 mm aluminum. The dose rate was 12.5 mGy/min and the total absorbed dosage was 75 mGy.

### Experimental designs

After 1 week of acclimatization, mice were randomly divided into five groups: control (*n =* 10); sham-irradiated (sham, mice were treated same as the LDR-treated mice except for irradiation, *n =* 10); LDR (a single dose of 75 mGy, *n =* 10), DOX (a single intraperitoneal injection of DOX at 7.5 mg/Kg, *n =* 20); LDR+DOX group (mice received LDR of 75 mGy as a pre-treatment and then 72 h later received DOX administration as in DOX group, *n =* 20). The general appearance, behavior, and survival rate were observed daily throughout the study. Five days after DOX injection, mice were anesthetized with sodium pentobarbital and subjected to ECG recording with Lab Chart7 Analysis Software (AD Instruments Inc., Australia) to measure arrhythmia. Mice were sacrificed for collecting blood and hearts. Then the blood samples that were allowed to clot and serum was separated by centrifugation at 3000 g for 10 min.

### Assessment of cardiotoxicity indices

Lactate dehydrogenase (LDH) activities were determined with commercially available LDH Kit according to the detail methods provided by the manufacturer’s protocol (Nanjing Jiancheng Bioengineering Institute, Nanjing, China).

### Histopathological examination

Heart specimens were fixed in 10% formalin and processed for paraffin sections of 4 μm thickness. Sections were stained with Hematoxylin and Eosin (H&E) and examined under a light microscope (Olympus BX-50 Olympus Corporation, Tokyo, Japan).

### Assessment of oxidative stress markers

Cardiac tissues were washed with cold saline. The heart tissue was prepared to 10% (10 g tissue/100 ml saline) homogenate for measuring SOD and GSH-Px activity and MDA content. The activities of SOD and GSH-Px and the content of MDA were determined with their respective assay kits, according to the manufacturer’s protocol (Nanjing Jiancheng Bioengineering Institute, Nanjing, China). In brief, the SOD activity was based on the xanthine oxidase method, the GSH-Px activity was determined according to dithio-bis-nitrobenzoic acid (DTNB) chromogenic reaction, and the MDA content was detected by thiobarbituric acid method.

### Terminal deoxynucleotidyl transferase biotin-d UTP nick end labeling (TUNEL)

Nuclear fragmentation was detected by TUNEL staining with an apoptosis detection kit according to the manufacturer’s protocol.

### Measurement of ROS

A fluorometric assay was used for the detection of ROS with the fluorescent probe (2′,7′-Dichlorodihydrofluorescein diacetate, DCFH-DA). DCFH-DA is hydrolyzed by cellular esterases to non-fluorescent 2′,7′-dichlorodihydrofluorescein (DCFH) and then oxidized to fluorescent 2′,7′-dichlorofluorescein (DCF) primarily by H_2_O_2_. Since DCFH might also be reactive toward a broad range of oxidizing reactions during intracellular oxidant stress. This probe is widely used to monitor the cellular redox processes. Therefore the generation of ROS in the myocardial cells was evaluated with this this assay based on a previously published method [52]. Briefly, single myocardial cells were incubated in serum-free DMEM. The homogenate was kept in ice, and centrifuged at 2000 rpm for 5 min. The myocardial cells were washed twice with PBS, and then resuspended at a concentration of 1 × 10^6^ cells/mL in serum-free DMEM containing DCFH-DA, before incubation at 37°C in a hatch box for 20 min. The fluorescent DCF generated in the cells was detected and analyzed with flow cytometry (BD FACSCalibur, San Jose, CA, USA).

### Measurement of mitochondrial membrane potential (∆ψm)

Changes in the ∆ψm were measured with a mitochondrial membrane potential assay kit with JC-1 (Beyotime, China), based on published method [52]. When JC-1 exists as J-aggregates in the mitochondrial matrix, it shows red fluorescence and when it exists as monomers in the cytoplasm, it shows green fluorescence. Therefore, the increase in the green fluorescence/red fluorescence intensity ratio, measured as ∆ψm, is an indication of increased mitochondrial permeability during mitochondrial depolarization. Briefly, method to isolate the cardiac cells and prepare the single cell suspension was same as those described above. The isolated myocardial cells were incubated with serum-free DMEM containing 5 g/mL JC-1 for 30 min. The stained cells were then rinsed twice with serum-free DMEM, fresh serum-free DMEM was added, and the cells were analyzed with flow cytometry with FACSCalibur as described previously [52].

### Immunohistochemistry

All specimens were fixed in 10% formalin, embedded in paraffin, cut into 4-μm-thick slides and processed sectioning. For immunohistochemical analysis of Caspase 9, Caspase 3, Bcl-2, and Bax expression, the slides were deparaffinized and rehydrated using standard techniques. Non-specific binding sites were blocked with complete serum at 37°C for 30 min. Then, the tissue sections were incubated with the Caspase 9, Caspase 3, Bcl-2, and Bax antibody (1:200) at 4°C overnight. The signal was visualized with peroxidase-labeled streptavidin-complexed DAB, and the sections were briefly counterstained with hematoxylin. Protein positive-staining exhibits a brown cytoplasmic and/or nuclear stain. Images were captured with the Olympus microscope. The positive cell density was assessed using Image-Pro Plus 6.0 software (Media Cybernetics, Bethesda, MD, USA), and the results are presented as mean optical density (MOD) values. The negative controls were handled in the same way except that PBS was applied in place of a primary antibody.

### Statistical analysis

All results were analyzed using SPSS software, version 19.0 for Windows (SPSS Inc., IL, SA). Data were presented as mean ± standard Error (SE). One-way ANOVA was used for multiple comparisons. *P* values < 0.05 were considered as statistically significant.
